# Prostate Cancer Susceptibility Loci Identified on Chromosome 12 in African Americans

**DOI:** 10.1371/journal.pone.0016044

**Published:** 2011-02-16

**Authors:** Carolina Bonilla, Stanley Hooker, Tshela Mason, Cathryn H. Bock, Rick A. Kittles

**Affiliations:** 1 School of Social and Community Medicine, University of Bristol, Bristol, Avon, United Kingdom; 2 Section of Genetic Medicine, Department of Medicine, The University of Chicago, Chicago, Illinois, United States of America; 3 National Human Genome Center at Howard University, Washington, D. C., United States of America; 4 Karmanos Cancer Institute, Wayne State University, Detroit, Michigan, United States of America; Ohio State University Medical Center, United States of America

## Abstract

Prostate cancer (PCa) is a complex disease that disproportionately affects African Americans and other individuals of African descent. A number of regions across the genome have been associated to PCa, most of them with moderate effects. A few studies have reported chromosomal changes on 12p and 12q that occur during the onset and development of PCa but to date no consistent association of the disease with chromosome 12 polymorphic variation has been identified. In order to unravel genetic risk factors that underlie PCa health disparities we investigated chromosome 12 using ancestry informative markers (AIMs), which allow us to distinguish genomic regions of European or West African origin, and tested them for association with PCa. Additional SNPs were genotyped in those areas where significant signals of association were detected. The strongest signal was discovered at the SNP rs12827748, located upstream of the *PAWR* gene, a tumor suppressor, which is amply expressed in the prostate. The most frequent allele in Europeans was the risk allele among African Americans. We also examined vitamin D related genes, *VDR* and *CYP27B1*, and found a significant association of PCa with the TaqI polymorphism (rs731236) in the former. Although our results warrant further investigation we have uncovered a genetic susceptibility factor for PCa in a likely candidate by means of an approach that takes advantage of the differential contribution of parental groups to an admixed population.

## Introduction

Prostate cancer (PCa) represents a major health burden to the US male population. In particular, African Americans are disproportionately affected by PCa, presenting with more advanced disease and worse prognosis than European Americans [1]. Reasons for the disparities include a number of genetic and environmental factors such as race, age, family history, socioeconomic status and access to health care [2].

Over the years there has been an accumulation of evidence in favour of a significant hereditary component in PCa susceptibility, however the search for PCa genes has yielded few consistent results. Recently, genome-wide linkage, association and admixture mapping scans have detected risk loci on chromosomes 2p15, 3p12, 6q25, 7q21, 8q24, 10q11, 11q13, 17q12, 17q24, 19q13, 22q13 and Xp11 [3] (see also GWAS catalog: http://www.genome.gov/gwastudies/), although polymorphic variants in these regions seem to account for only a small proportion of the observed genetic predisposition to PCa.

To determine whether the health inequalities between African Americans and European Americans can be partially explained by differences in genetic background we searched for alleles with large frequency differences between these populations that also have an incidence on susceptibility to PCa.

In a preliminary screening of 21 ancestry informative markers (AIMs) on chromosome 12 in 368 African American PCa cases and controls we found an indication of association across an extended region (from 23 Mb to 64 Mb). We increased the sample size and coverage of the chromosome with additional AIMs and SNPs in an attempt to narrow the region of interest and if possible, identify the causal variant. Special consideration was given to the vitamin D receptor (*VDR*, OMIM*601769) gene, located on chromosome 12q13.11, as it is a likely candidate and previous reports have revealed an association between polymorphisms in this gene and PCa [4].

## Materials and Methods

### Subjects

Five hundred and fifty unrelated African American cases and controls were included in the study. Recruitment for both patients and healthy controls took place at Howard University Hospital in Washington, D.C, through the Division of Urology and prostate cancer screening programs.

PSA levels and other clinical assessments were determined for all PCa cases and all controls. Prostate cancer patients did not have more than one first degree relative with PCa. All controls had a normal digital rectal examination.

Disease aggressiveness was defined based on TNM stage and Gleason grade. Low aggressiveness was determined by a T category<T2c and a Gleason grade <7. Conversely, high aggressiveness was characterized by a T category ≥T2c and a Gleason grade ≥7. The study was approved by Howard University and the University of Chicago hospitals Institutional Review Board and by all participants, who provided written informed consent.

Genomic DNA was obtained from peripheral blood samples using standard techniques.

### Genotyping

A set of 124 ancestry informative markers (AIMs) located on chromosome 12, most of which were selected from a published admixture map tailored for African Americans [5], were genotyped in cases and controls and utilized to estimate local ancestry. In addition, individual admixture proportions were estimated using a set of 103 genome-wide AIMs chosen from the same panel. Individuals belonging to each parental population, i.e. HapMap Yoruba (YRI) and CEPH (CEU), were also typed for these markers.

In addition, we genotyped fifteen SNPs in two genes that are involved in vitamin D metabolism, eleven on the vitamin D receptor (*VDR*) and four on *CYP27B1* (OMIM*609506).

Finally, genotypes for a further set of 55 SNPs on chromosome 12 were collected and analysed in an attempt to circumscribe the associated regions to specific genes.

The Illumina BeadLab platform [6] was used to genotype all AIMs in this project. *VDR*, *CYP27B1* and all SNPs used for finer mapping were assayed with the Sequenom MassArray platform [7].

All physical positions and SNP details reported in the current study were based on NCBI genome build 36 and dbSNP build 130.

### Data analysis

The program STRUCTURE v2.2 [8] was run to estimate individual and locus-specific ancestry. For our analysis the number of parental populations was set at two. Runs consisted basically of 30000 burn-ins and 70000 iterations, and 5000 admixture burn-ins when necessary. To collect data on site-by-site (local) ancestry we used the linkage model, whereas a model that uses population information from parental individuals' genotypes was selected to obtain individual ancestry proportions.

Linkage disequilibrium (LD) patterns along chromosome 12 were examined with the program Haploview v4.2 [9]. Selection of tagSNPs was also done with Haploview using the YRI as the reference population, pairwise tagging and a r^2^ threshold of 0.8.

Binary logistic regression was used to estimate odds ratios (OR) and 95% confidence intervals (CI) for the association of individual markers and haplotypes with PCa with adjustment for age, individual and local ancestry, via the programs SPSS v15 and PLINK 1.07 [10]. Power calculations were performed with the program Genetic Power Calculator [11].

Functional *in silico* analysis of gene variants to identify changes in transcription factor binding sites, 5′UTR and 3′UTR motifs, and conservation across species was carried out using the programs included in the GenEpi toolbox [12].

## Results

A total of 669 individuals were entered in the analysis, including 253 African American PCa patients, 297 African American controls, 59 West Africans (HapMap YRI) and 60 Europeans (HapMap CEU). Clinical and demographic characteristics of the African American sample are presented in Table 1.

**Table 1:** Sample description.

	cases	controls	p-value
N	253	297	
mean age (sd)	65.9 (9.3)	58.9 (11.0)	<0.001
mean PSA (sd)	85.8 (393.9)	4.9 (15.0)	0.002
mean African ancestry (% ± sd)	79.0 (16.3)	77.2 (18.2)	0.24
**PCa aggressiveness**			
low (%)	82 (42.3)	-	
high (%)	112 (57.7)	-	

After eliminating markers with more than 50% missing genotypes, less than 5% minor allele frequency (MAF), out of Hardy-Weinberg equilibrium in controls (p<0.0005), closely linked to others (<0.1 cM intermarker distance), 76 chromosome 12 AIMs and 88 genome-wide AIMs were analyzed (Tables S1 and S2).

Based on the power analyses we had on average ∼70% power (36–83%) to detect a genotype relative risk of 1.5 at an alpha level of 0.05 for variants with a minor allele frequency ≥5%, assuming the marker is in complete LD with the causal variant and a prevalence of PCa in African Americans of 2.5%. If all variants were considered, power decreased to ∼60% (8–83%) due to the fact that there is limited power to detect the effects of variants with frequencies below 5% (22% power on average), see Table S3.

### Ancestry and population structure

Using the set of genome-wide AIMs, mean allele frequency difference (delta) between parental populations was found to be 74%, extending from 55% to 96% (Table S2). STRUCTURE runs established two as the most probable number of clusters within African Americans, irrespective of status, indicating the presence of population stratification. Average European individual ancestry (± sd) was 22±17% for the whole sample, similar to previously reported estimates for this population [13]. PCa cases had on average higher West African ancestry than controls (79% vs 77%) although the difference was not significant (Table 1). Patients were significantly older than controls (p<0.001, Table 1).

### Association with disease status

Our initial analysis using 242 cases and 126 controls found that three out of 21 AIMs were significantly associated with disease status after adjustment for age and individual ancestry (p<0.05, Table 2), while another AIM was close to significance (rs1963562, p = 0.07).

**Table 2:** Initial analysis of chromosome 12 AIMs.

SNP id	chromosomal position (bp)	gene	location in gene	major/minor alleles^a^	delta^b^	cases	controls	p-value^c^
rs989278	23773750	SOX5	intron 7	C/G	0.08	0.34	0.33	0.48
**rs1993973**	**26201374**	**-**		**G/A**	**0.63**	**0.45**	**0.38**	**0.004**
rs1027089	26362337	-		C/G	0.33	0.16	0.14	0.82
rs895964	26749333	ITPR2	intron 10	G/A	0.36	0.21	0.26	0.98
rs1010096	27735497	PPFIBP1	intron 28	C/T	0.32	0.44	0.43	0.11
rs1561131	29199198	-		C/T	0.39	0.52	0.48	0.40
**rs1843321**	**30245161**	**-**		**C/T**	**0.57**	**0.37**	**0.41**	**0.04**
rs1963562	36949163	-		C/T	0.39	0.11	0.10	0.07
rs870431	37239340	-		A/G	0.55	0.15	0.14	0.64
rs278906	39704904	CNTN1	intron 21	C/T	0.24	0.17	0.23	0.34
rs180438	45473527	SLC38A4	intron 2	G/A	0.67	0.23	0.24	0.11
rs739857	46254553	-		A/C	0.40	0.14	0.11	0.52
rs1235153	46396592	P11	intron 5	C/T	0.05	0.01	0.00	0.64
rs615382	48699178	RACGAP1	intron 3	C/A	0.73	0.32	0.28	0.11
rs303810	50449515	SCN8A	intron 18	T/C	0.49	0.48	0.47	0.96
**rs585224**	**51209139**	**-**		**A/G**	**0.51**	**0.28**	**0.23**	**0.004**
rs1683151	52232486	ATF7	intron 5	C/G	0.51	0.11	0.09	0.99
rs1867299	52616242	-		T/C	0.50	0.30	0.33	0.92
rs1078109	56272633	PIP4K2C	intron 2	G/A	0.07	0.01	0.01	0.27
rs1157239	63814994	-		G/A	0.52	0.32	0.27	0.80
rs343087	64547191	HMGA2	intron 4	A/G	0.78	0.19	0.16	0.85

a major/minor allele assignment based on frequencies found in West Africans. Minor allele frequency is given.

b delta = allele frequency difference between West African and European populations.

c p-value after adjustment for age and individual ancestry. **Bold**: SNPs associated with PCa, p<0.05.

In order to further investigate these association signals we expanded the chromosome 12 AIM set with 75 additional polymorphisms and increased the sample size to a total of 550 individuals. Mean allele frequency difference between West Africans and Europeans for this enlarged set of AIMs was 70% (range: 24%–95%), and the average distance between markers was 1.25 Mb (Table S1). Coverage of the chromosome extended from 0.2 Mb to 131.2 Mb. Very few markers of this AIM panel are in LD across the region, and those that do show low R^2^ values. This is expected to some extent as variants were selected to ensure the absence of LD in the parental populations.

Association with PCa was uncovered for ten new markers at an alpha level of 0.05, adjusting for age and individual ancestry, although none achieved significance if applying a Bonferroni correction for multiple testing (p<0.0007, Table S1). Interestingly, for all loci, the most frequent allele among Europeans was also the allele confering risk for PCa.

We selected 55 extra SNPs within the associated regions and neighbouring candidate genes to try to pinpoint the source of the association signal. Preference was given to tagSNPs as defined by the program Haploview in the YRI population. This was done in two stages, 20 SNPs were typed and analysed first and, based on these and earlier results, 35 more variants were added, particularly in the genes *TMTC1*, *HMGA2* (OMIM*600698) and *PAWR* (OMIM*601936), which displayed the strongest association with PCa (Tables S1 and S4). Six SNPs were invariant in this population whilst three were out of Hardy-Weinberg equilibrium (p<0.002), therefore 46 polymorphisms were included in the final analysis, of which four were significantly associated with PCa (one in *HMGA2* and three in *PAWR*). We did not pursue the *HMGA2* association any further since rs17179670 is an intronic variant, is the only polymorphism associated to disease within this gene and the association signal is not particularly strong (p = 0.03).

### The PAWR gene region

Following all rounds of analysis rs12827748, located upstream of the *PAWR* gene, remained the most significantly associated SNP, even after adjustment for age and individual ancestry (p<0.01). This SNP shows a large frequency difference between European and West African populations (C allele: 0.63 vs 0.01, respectively), whereas the frequency of the same allele in PCa cases is 0.18 compared to 0.11 in controls (Table S4). To eliminate added confounding by ancestry we included local ancestry estimates in the regression model.

Local ancestry at rs12827748 was assessed using the AIM rs10778691, which was the closest one to rs12827748 (distant ∼130 kb), and revealed that the probability of two European chromosomes at this locus was greater in cases than in controls, although the result was not significant (8.3% vs 7.6%, p = 0.74). Additional adjustment for local ancestry did not eliminate the significance of the association between rs12827748 and PCa (OR 1.6; 95% CI, 1.1–2.4; p = 0.02, Table 3).

**Table 3:** Odds ratios and 95% confidence intervals of *PAWR* and *VDR* SNPs significantly associated with prostate cancer.

PAWR	risk allele	OR	95% CI	p-value	adj OR	95% CI	p-value
rs8176908	T	1.7	1.1–2.6	0.03	1.7	1.1–2.8	0.03
rs8176882	C	2.2	1.0–4.8	0.04	2.8	1.2–6.3	0.02
rs12827748	C	1.6	1.1–2.2	0.01	1.6	1.1–2.4	0.02

VDR	risk allele	OR	95% CI	p-value	adj OR	95% CI	p-value
rs731236	C	1.3	1.0–1.8	0.04	1.3	1.0–1.7	0.11
rs7975128	A	1.4	1.1–1.8	0.02	1.3	1.0–1.7	0.07

Odds ratios per each additional risk allele, adjusted by age, individual and local ancestry for *PAWR*, and age and individual ancestry for *VDR*. rs8176908 and rs8176882 were tested as GG vs GT/TT and GG vs GC/CC, respectively, as they were rare SNPs.

Two other *PAWR* SNPs were also significant (rs8176908, rs8176882, p<0.05, Tables 3 and S4), the minor alleles are completely absent in European populations and have a lower than 10% frequency among West Africans. Yet, when all three markers are included in the logistic regression, with age, individual and local ancestry as covariates, only rs12827748 exhibits a significant association (p = 0.03). Linkage disequilibrium is strong between rs8176908 and rs8176882 but weaker between any of these and rs12827748 (data not shown).

Haplotypes constructed using the three *PAWR* polymorphisms showed a strong protective effect of the TGG haplotype (p = 1.7×10^−4^) whereas the haplotypes that included all European alleles (i.e. alleles that have a higher frequency in European than in West African populations, CGG) or all West African alleles (TCT) significantly increased the risk of disease (Table 4). Only the TGG haplotype remained significant after correction for multiple testing by permutation analysis.

**Table 4:** *PAWR* haplotypes and prostate cancer risk.

	haplotype	cases	controls	OR	95% CI	p-value	adj OR	95% CI	p-value
PAWR-H1	TGG	0.73	0.83	0.6	0.4–0.8	7.8E-05	0.5	0.4–0.7	1.7E-04
PAWR-H2	CGG	0.18	0.11	1.6	1.1–2.2	0.01	1.7	1.1–2.5	0.01
PAWR-H3	TGT	0.06	0.04	1.5	0.8–2.6	0.18	1.4	0.8–2.6	0.24
PAWR-H4	TCT	0.03	0.01	2.6	1.1–6.1	0.03	3.0	1.2–7.6	0.02

SNPs: rs12827748–rs8176882–rs8176908. Omnibus test: p = 0.001. Odds ratios adjusted by age, individual and local ancestry.

We assessed *in silico* the impact that rs12827748 may have on gene function and found no disruption of transcription factor binding sites or other regulatory effects. This is an intergenic SNP that lies between *PAWR* and *PPP1R12A* and it may be in linkage disequilibrium with functional variants in either gene, although *PAWR* as a tumor suppressor is a more likely candidate. Nevertheless, LD patterns in HapMap populations CEU and YRI show that rs12827748 can be found within a large block that includes all of *PAWR* and its upstream region with a clearcut separation from the block that contains *PPP1R12A*.

As expected, we noted that carriers of the rs12827748 C allele displayed significantly higher mean European ancestry than individuals with the TT genotype (0.30 vs 0.20, p<0.005), yet there were no differences in ancestry between cases and controls within each genotype group. However, if individuals are selected based on local ancestry levels, for instance by restricting the sample to those individuals with a higher than 95% probability of having two European chromosomes at rs10778691 (N = 32) or to those carrying the rs12827748 CC genotype (N = 21), cases show a significantly elevated global West African ancestry (54–66% vs 32–34% in controls), although it is still low with respect to the average of the unselected sample (78%). In spite of sample sizes being quite small the differences are significant (p = 0.03 and 0.01). This suggests that there likely are additional factors inherited from the West African ancestor that predispose to PCa.

### Vitamin D related genes: VDR and CYP27B1

The active form of vitamin D (1,25(OH)_2_D_3_) reduces proliferation and promotes differentiation, and has been implicated in cancers of the colon, breast and prostate. At the same time it has been reported that individuals of West African descent have approximately twofold lower levels of serum vitamin D (25(OH)D, the major circulating metabolite) than those of predominantly European ancestry [14], which may imply less protection from cancer for the former. It has been suggested that polymorphic variation within genes in the vitamin D pathway, such as *VDR*, may contribute to the observed disparities in PCa rates between populations of different origins.

Several studies have examined variation in *VDR* in search for an association with different types of cancer. There are six commonly studied polymorphisms in *VDR*: Cdx2 variant in the promoter (rs11568820), FokI on exon 2 (rs2228570), BsmI on intron 8 (1544410), ApaI also on intron 8 (rs7975232), TaqI on exon 9 (rs731236), and the poly-A mononucleotide repeat at the 3′UTR of the gene. TaqI is in strong LD with the other markers in the region (BsmI, ApaI and the poly-A microsatellite). Polymorphisms in the 5′ regulatory region are known to affect the transcriptional activity of the gene whereas polymorphisms in the 3′UTR have been linked to the stability of mRNA [15]. Results for PCa have been inconsistent with some studies showing positive associations and others postulating no effects [16]–[19]. An association with advanced PCa has also been reported, i.e. Gleason grade ≥7 [20].

Since two of the genes in the vitamin D pathway, namely *VDR* and *CYP27B1*, are located on chromosome 12, we examined them more closely by typing a few SNPs in them. *CYP27B1* encodes the enzyme 1-α-hydroxylase which catalyzes the conversion of 25(OH)D into 1,25(OH)_2_D_3._ Polymorphic variants in this gene have been examined but found not to be associated with PCa [21]. Details of the SNPs typed are given in Table 5. Association with disease was seen for two closely linked *VDR* SNPs, rs731236 (TaqI) and rs7975128 (p<0.05, Tables 3 and 5), and their haplotypes (Table 6). However, adjustment by age and individual ancestry removed the significance of the associations. No effect of *CYP27B1* variants on PCa risk was evident in this study.

**Table 5:** SNPs typed in vitamin D pathway genes located on chromosome 12.

SNP id	chromosomal position (bp)	gene	major/minor alleles^a^	cases	controls	crude p-value	gene region
**rs731236**	**46525024**	**VDR**	**T/C**	**0.33**	**0.27**	**0.04**	**I352I (TaqI)**
**rs7975128**	**46532095**	**VDR**	**G/A**	**0.33**	**0.27**	**0.02**	**intron 8**
rs2229828	46537572	VDR	C/T	0.02	0.03	0.29	S148S
rs2248098	46539623	VDR	C/T	0.45	0.41	0.27	intron
rs2228570	46559162	VDR	G/A	0.20	0.20	0.80	T1M (FokI)
rs2254210	46559981	VDR	G/A	0.34	0.34	1.00	intron
rs11574038	46563420	VDR	G/A	0.04	0.03	0.57	intron
rs1989969	46564277	VDR	C/T	0.43	0.43	0.85	intron
rs12581281	46581400	VDR	C/T	0.03	0.05	0.15	intron
rs12721377	46581618	VDR	A/G	0.03	0.02	0.22	intron
rs11568820	46588812	VDR	A/G	0.21	0.22	0.91	promoter (Cdx2)
rs4646537	56443548	CYP27B1	A/C	0.07	0.06	0.47	intron
rs3782130	56448165	CYP27B1	C/G	0.10	0.12	0.38	upstream
rs10877012	56448352	CYP27B1	G/T	0.11	0.12	0.37	upstream
rs703842	56449006	CYP27B1/METTL1	T/C	0.29	0.31	0.55	upstream

a major/minor allele assignment based on frequencies found in West Africans. Minor allele frequency is given. **Bold**: SNPs associated with PCa, p<0.05.

**Table 6:** *VDR* haplotypes and prostate cancer risk.

	haplotype	cases	controls	OR	95% CI	p-value	adj OR	95% CI	p-value
VDR-H1	TG	0.56	0.64	0.7	0.6–1.0	0.02	0.8	0.6–1.1	0.12
VDR-H2	CA	0.23	0.18	1.4	1.0–1.9	0.06	1.3	0.9–1.9	0.11
VDR-H3	TA	0.11	0.10	1.2	0.8–1.8	0.42	1.1	0.7–1.7	0.75
VDR-H4	CG	0.10	0.10	1.1	0.7–1.8	0.67	1.0	0.6–1.7	0.94

SNPs: rs731236–rs7975128. Omnibus test: p = 0.15. Odds ratios adjusted by age and individual ancestry.

## Discussion

We have screened chromosome 12 in the search for alleles that predispose African Americans for the onset and development of PCa and which may partially account for the differences in incidence and mortality between African Americans and European Americans. After carefully controlling for the effects of individual and local ancestry we identified a polymorphism (rs12827748) located upstream of the *PAWR* gene that significantly increased susceptibility for PCa. The C allele, which confers risk, is rare in West Africans but is the major allele in European populations. Two additional *PAWR* SNPs (one intronic, one synonymous) were also associated with PCa in the single marker analysis but the inclusion of the three polymorphisms in the regression model eliminated all significance except for rs12827748. However, since the latter two variants are only present in populations of West African descent they may also represent PCa risk factors that because of their lower frequencies are not detected as independently significant signals. There is then the possibility that this region harbors susceptibility factors from European as well as West African origin, and in an admixed population such as African Americans both of them could be at work.

It is also interesting to note that all of the associated SNPs were in fact low frequency variants with frequencies around or below 10% in controls. Odds ratios corresponding to these variants ranged from 1.6 to 2.8, higher than those usually reported in genome-wide association studies. Because of the relatively small size of our study there is not enough power to detect the effect of rare variants (i.e. those with frequencies lower than 1%) and therefore cannot disregard them as potential PCa causative factors. Similarly, the importance of variants with frequencies below 5% may have been underestimated because of inadequate power.

The *PAWR* or prostate apoptosis response gene is located on chromosome 12q21 and is highly expressed in the prostate. It promotes apoptosis and causes tumor regression, and its downregulation is apparent during tumorigenesis [22]. However, loss of *PAWR* is not enough to cause PCa in *PAWR* null mice but does so when coupled with concomitant *PTEN* heterozygosity [23]. Our results suggest that *PAWR* may be a low penetrance PCa susceptibility gene and deserves further examination in other populations and with respect to other PCa related phenotypes. The relevance of these findings will, however, only be determined by their replication in subsequent studies as we cannot ignore the fact that studies with small sample sizes are at a higher risk of reporting false positive associations [24].

The limited number of cases and controls also affected our ability to conduct a formal admixture mapping study on chromosome 12 as there is reduced power to detect a less than 2-fold risk due to ancestry [25].

We detected a marginally significant association of PCa with two SNPs in the *VDR* gene, including the TaqI polymorphism. Contrary to what has been found in a recent meta-analysis the minor allele (C = t) at TaqI was the risk allele in our study, although the meta-analysis significant association was identified by pooling together European, African and Asian populations [4]. However, a closer look at the data stratified by ethnicity indicates that whereas the C allele appears to be protective in Europeans and Asians it seems to confer risk in populations of African descent [4]. Most of the studies that analyzed TaqI in Europeans did not find a significant effect or reported that the major (T) allele increased susceptibility to PCa [26] [27]. On the other hand, studies of VDR polymorphisms in African Americans with PCa are fairly limited, the majority with small sample sizes and consequently inconclusive with respect to the role of TaqI [26].

Finally, it is important to emphasize that the careful genome-wide evaluation of the ancestral origins of an admixed population such as African Americans represents a valuable means of unraveling the genetic risk factors that are likely to contribute to the existence of health disparities.

## Supporting Information

Table S1 List of 76 ancestry informative markers (AIMs) distributed along chromosome 12 used to estimate local ancestry and detect association with prostate cancer.
(DOC)

Table S2 Genome-wide ancestry informative markers (AIMs) used in the estimation of individual ancestry proportions in African American prostate cancer cases and controls.
(DOC)

Table S3 Estimated power to detect effects of chromosome 12 SNPs.
(XLSX)

Table S4 Fifty-five SNPs typed in previously associated or candidate chromosome 12 regions.
(DOC)
