# Supplementary material for: Prostate Cancer Susceptibility Loci Identified on Chromosome 12 in African Americans
Source: PLoS One. 2011 Feb 16;6(2):e16044. doi: 10.1371/journal.pone.0016044 (PMC3040176; doi:10.1371/journal.pone.0016044)
Supplement: Table S1 — List of 76 ancestry informative markers (AIMs) distributed along chromosome 12 used to estimate local ancestry and detect association with prostate cancer. (DOC) [file pone.0016044.s001.doc]

| Table S1. List of 76 ancestry informative markers (AIMs) distributed along chromosome 12 used to estimate local ancestry and detect association with prostate cancer. | | | | | | | | | |
| --- | --- | --- | --- | --- | --- | --- | --- | --- | --- |
| **SNP id** | **chromosomal position (bp)** | **major/minor alleles^a^** | **gene** | **Africans** | **Europeans** | **delta^b^** | **cases** | **controls** | **p-value^c^** |
| rs124440 | 202923 | G/C | SLC6A13 | 0.78 | 0.16 | 0.62 | 0.68 | 0.67 | 0.58 |
| rs7967165 | 950268 | T/C |  | 0.92 | 0.18 | 0.74 | 0.74 | 0.72 | 0.66 |
| rs11062437 | 2947460 | G/A | TEAD4 | 0.93 | 0.16 | 0.77 | 0.74 | 0.75 | 0.62 |
| rs10744614 | 3425163 | A/C |  | 0.89 | 0.18 | 0.71 | 0.76 | 0.75 | 0.72 |
| rs11063488 | 5057090 | G/T |  | 0.89 | 0.31 | 0.57 | 0.78 | 0.80 | 0.15 |
| rs7303229 | 5540711 | A/T | TMEM16B | 0.90 | 0.27 | 0.63 | 0.72 | 0.69 | 0.63 |
| rs3782711 | 6053442 | C/T | VWF | 0.82 | 0.14 | 0.69 | 0.68 | 0.69 | 0.43 |
| rs11064432 | 6839002 | G/C | USP5 | 0.89 | 0.04 | 0.85 | 0.70 | 0.69 | 0.69 |
| rs10840686 | 8152917 | G/C |  | 0.91 | 0.29 | 0.62 | 0.80 | 0.78 | 0.83 |
| rs10845099 | 10312069 | G/C |  | 0.80 | 0.09 | 0.71 | 0.64 | 0.63 | 0.55 |
| rs1861482 | 11724925 | A/C | ETV6 | 0.87 | 0.13 | 0.74 | 0.77 | 0.76 | 0.83 |
| rs10772518 | 12017303 | C/G |  | 0.86 | 0.16 | 0.70 | 0.70 | 0.70 | 0.78 |
| rs850918 | 13033441 | C/G | HEBP1 | 0.63 | 0.12 | 0.24 | 0.64 | 0.61 | 0.71 |
| rs7310891 | 14707830 | G/A | GUCY2C | 0.72 | 0.01 | 0.71 | 0.56 | 0.58 | 0.34 |
| rs984303 | 15641658 | G/A | PTPRO | 0.79 | 0.02 | 0.78 | 0.64 | 0.63 | 0.77 |
| rs7967366 | 19079208 | T/C |  | 0.86 | 0.13 | 0.72 | 0.70 | 0.72 | 0.17 |
| rs10841722 | 21057358 | A/G |  | 0.91 | 0.03 | 0.88 | 0.67 | 0.69 | 0.20 |
| rs4350408 | 22043980 | A/C |  | 0.81 | 0.05 | 0.76 | 0.62 | 0.64 | 0.52 |
| **rs4514479** | **23300566** | **G/C** |  | **0.93** | **0.23** | **0.70** | **0.71** | **0.78** | **0.01** |
| rs7307747 | 25389896 | C/T |  | 0.82 | 0.07 | 0.76 | 0.62 | 0.64 | 0.28 |
| **rs7975017** | **26320060** | **T/C** |  | **0.91** | **0.18** | **0.73** | **0.72** | **0.76** | **0.03** |
| rs7963493 | 26526342 | C/T | ITPR2 | 0.91 | 0.22 | 0.69 | 0.71 | 0.74 | 0.09 |
| **rs299478** | **29598519** | **T/C** | **TMTC1** | **0.79** | **0.10** | **0.69** | **0.58** | **0.66** | **0.004** |
| rs4931655 | 32949329 | G/A |  | 0.86 | 0.06 | 0.80 | 0.58 | 0.62 | 0.16 |
| rs7978988 | 34288146 | T/C |  | 0.90 | 0.02 | 0.88 | 0.65 | 0.65 | 0.68 |
| rs11182432 | 42855115 | A/C | TMEM117 | 0.80 | 0.10 | 0.70 | 0.60 | 0.63 | 0.09 |
| **rs2471601** | **45234926** | **A/C** |  | **0.88** | **0.10** | **0.78** | **0.69** | **0.73** | **0.05** |
| rs7970314 | 46594441 | G/A |  | 0.99 | 0.20 | 0.79 | 0.81 | 0.81 | 0.48 |
| rs615382^d^ | 48699178 | C/A | RACGAP1 | 0.89 | 0.08 | 0.82 | 0.71 | 0.72 | 0.11 |
| **rs7306523** | **51680231** | **A/G** |  | **0.98** | **0.16** | **0.82** | **0.78** | **0.83** | **0.001** |
| rs11171526 | 54192728 | A/T |  | 0.79 | 0.04 | 0.75 | 0.61 | 0.62 | 0.61 |
| rs10876851 | 54471828 | A/C | CIP29 | 0.84 | 0.05 | 0.79 | 0.64 | 0.67 | 0.31 |
| rs899653 | 56170068 | G/A | MARS | 0.87 | 0.13 | 0.73 | 0.70 | 0.72 | 0.53 |
| rs7132009 | 58873307 | A/G |  | 0.74 | 0.04 | 0.70 | 0.54 | 0.56 | 0.21 |
| rs35445530 | 63541720 | A/C | TBC1D30 | 0.78 | 0.04 | 0.74 | 0.58 | 0.63 | 0.10 |
| **rs11175885** | **64400897** | **A/G** |  | **0.87** | **n/a** | **n/a** | **0.71** | **0.76** | **0.001** |
| **rs10878561** | **65791320** | **A/G** |  | **0.94** | **0.15** | **0.79** | **0.68** | **0.75** | **0.001** |
| rs918003 | 66562762 | G/A |  | 0.81 | 0.15 | 0.66 | 0.64 | 0.67 | 0.08 |
| rs10784930 | 70374571 | T/C | TMEM19 | 0.93 | 0.22 | 0.71 | 0.78 | 0.80 | 0.16 |
| rs460406 | 71298182 | A/G | TRHDE | 0.91 | 0.21 | 0.70 | 0.80 | 0.79 | 0.58 |
| **rs7967984** | **74600665** | **T/A** |  | **0.98** | **0.28** | **0.69** | **0.77** | **0.82** | **0.01** |
| rs1402317 | 76753496 | C/T | NAV3 | 0.89 | 0.13 | 0.77 | 0.70 | 0.73 | 0.34 |
| rs7138514 | 78318689 | G/T | SYT1 | 0.86 | 0.03 | 0.83 | 0.62 | 0.65 | 0.26 |
| rs10778691 | 78744019 | T/G | PPP1R12A | 0.99 | 0.11 | 0.87 | 0.77 | 0.79 | 0.29 |
| rs11115459 | 81768245 | C/T | TMTC2 | 0.91 | 0.05 | 0.87 | 0.70 | 0.72 | 0.22 |
| **rs9651975** | **84174670** | **A/G** |  | **0.82** | **0.08** | **0.74** | **0.65** | **0.69** | **0.02** |
| rs388693 | 87696843 | A/G |  | 0.91 | 0.08 | 0.83 | 0.69 | 0.66 | 0.54 |
| rs2731232 | 89098242 | G/C |  | 0.97 | 0.11 | 0.86 | 0.78 | 0.75 | 0.84 |
| rs10859541 | 92534318 | G/A |  | 0.80 | 0.07 | 0.73 | 0.65 | 0.64 | 0.76 |
| rs7975526 | 93566970 | C/G | TMCC3 | 0.79 | 0.23 | 0.56 | 0.63 | 0.59 | 0.35 |
| rs201392 | 97763262 | A/T | ANKS1B | 0.82 | 0.15 | 0.66 | 0.67 | 0.62 | 0.17 |
| rs11110411 | 99442611 | C/T | NR1H4 | 0.61 | 0.01 | 0.60 | 0.46 | 0.42 | 0.44 |
| rs2242138 | 100273035 | C/A | UTP20 | 0.67 | 0.02 | 0.65 | 0.53 | 0.51 | 0.85 |
| rs12820008 | 101676159 | A/C |  | 0.95 | 0.29 | 0.65 | 0.79 | 0.78 | 0.91 |
| rs6539244 | 104875232 | C/T |  | 0.77 | 0.02 | 0.75 | 0.61 | 0.61 | 0.86 |
| rs916682 | 110183529 | G/A | CUTL2 | 0.95 | n/a | 0.95 | 0.81 | 0.82 | 0.37 |
| rs11066430 | 111753980 | C/T | RPH3A | 0.68 | 0.04 | 0.64 | 0.48 | 0.51 | 0.12 |
| rs4767252 | 113672107 | A/C |  | 0.92 | 0.31 | 0.61 | 0.75 | 0.77 | 0.32 |
| rs1549338 | 114646999 | A/G |  | 0.84 | 0.24 | 0.61 | 0.76 | 0.77 | 0.74 |
| **rs7969190** | **115455075** | **C/T** | **FLJ42957** | **0.74** | **0.12** | **0.62** | **0.58** | **0.63** | **0.02** |
| rs4767461 | 115771364 | C/T | TMEM118 | 0.99 | 0.30 | 0.69 | 0.84 | 0.85 | 0.59 |
| rs628644 | 116589307 | A/G | KSR2 | 1.00 | 0.29 | 0.70 | 0.83 | 0.84 | 0.45 |
| rs280591 | 118775101 | C/T | CIT | 0.71 | 0.03 | 0.69 | 0.54 | 0.56 | 0.99 |
| rs4077055 | 120038806 | A/G |  | 0.77 | 0.07 | 0.70 | 0.57 | 0.56 | 0.73 |
| rs6486783 | 120858205 | A/G | WDR66 | 0.68 | 0.06 | 0.62 | 0.52 | 0.56 | 0.45 |
| rs1051431 | 122211756 | G/A | MPHOSPH9 | 0.97 | 0.23 | 0.73 | 0.76 | 0.74 | 0.70 |
| rs1070544 | 123742318 | C/A |  | 0.83 | 0.02 | 0.81 | 0.63 | 0.61 | 0.74 |
| rs2615667 | 124569401 | T/C | TMEM132B | 0.83 | 0.12 | 0.72 | 0.66 | 0.64 | 0.33 |
| rs12304312 | 125415094 | G/A |  | 0.69 | 0.01 | 0.68 | 0.51 | 0.51 | 0.98 |
| rs12370733 | 126421324 | T/A |  | 0.71 | 0.07 | 0.65 | 0.60 | 0.58 | 0.80 |
| rs1560545 | 126870218 | G/A |  | 0.88 | 0.21 | 0.67 | 0.72 | 0.72 | 0.54 |
| rs1709704 | 127386467 | G/C | TMEM132C | 0.78 | 0.00 | 0.78 | 0.59 | 0.61 | 0.28 |
| rs1486638 | 128151364 | C/G | TMEM132D | 0.58 | 0.02 | 0.56 | 0.50 | 0.50 | 0.76 |
| rs4759479 | 129541026 | A/G | RIMBP2 | 0.65 | 0.00 | 0.65 | 0.48 | 0.45 | 0.31 |
| rs7397250 | 130615744 | T/C |  | 0.90 | 0.21 | 0.69 | 0.74 | 0.72 | 0.60 |
| rs11835531 | 131087534 | G/A | EP400 | 0.90 | 0.12 | 0.78 | 0.76 | 0.74 | 0.92 |
| ^a^major/minor allele assignment based on frequencies found in West Africans. Major allele frequency is given. **^b^**delta: allele frequency difference between West African and European populations. ^c^p-values have been adjusted for age and individual ancestry. ^d^SNP present in initial set of 21 AIMs. **Bold**: SNPs associated with PCa, p<0.05. | | | | | | | | | |
