# Supplementary material for: Prostate Cancer Susceptibility Loci Identified on Chromosome 12 in African Americans
Source: PLoS One. 2011 Feb 16;6(2):e16044. doi: 10.1371/journal.pone.0016044 (PMC3040176; doi:10.1371/journal.pone.0016044)
Supplement: Table S2 — Genome-wide ancestry informative markers (AIMs) used in the estimation of individual ancestry proportions in African American prostate cancer cases and controls. (DOC) [file pone.0016044.s002.doc]

| **Table S2.** Genomewide ancestry informative markers (AIMs) used in the estimation of individual ancestry proportions in African American prostate cancer cases and controls. | | | | | | | |
| --- | --- | --- | --- | --- | --- | --- | --- |
| **SNP id** | **chromosome** | **chromosomal position (bp)** | **major/minor alleles^a^** | **Africans** | **Europeans** | **delta^b^** | **p-value^c^** |
| rs4920311 | 1 | 18436578 | G/C | 0.92 | 0.11 | 0.81 | 0.56 |
| rs667016 | 1 | 47783923 | T/A | 0.90 | 0.14 | 0.76 | 0.77 |
| rs11210458 | 1 | 74650713 | A/G | 0.93 | 0.31 | 0.62 | 0.37 |
| rs4311917 | 1 | 107527266 | C/A | 0.81 | 0.18 | 0.63 | 0.86 |
| rs2768744 | 1 | 153685222 | G/A | 0.98 | 0.14 | 0.84 | 0.53 |
| rs726777 | 1 | 184139446 | T/A | 0.92 | 0.20 | 0.72 | 0.67 |
| rs979698 | 1 | 207764070 | T/C | 0.91 | 0.18 | 0.73 | 0.77 |
| rs630101 | 1 | 234839039 | G/A | 0.98 | 0.18 | 0.80 | 0.19 |
| rs2380549 | 2 | 14025329 | A/G | 0.85 | 0.06 | 0.79 | 0.57 |
| rs13385952 | 2 | 41384902 | C/T | 0.90 | 0.13 | 0.77 | 0.78 |
| rs11890727 | 2 | 114383964 | G/C | 0.79 | 0.10 | 0.69 | 0.06 |
| rs1979038 | 2 | 148927368 | A/G | 0.79 | 0.14 | 0.65 | 0.76 |
| rs2271767 | 2 | 191987572 | T/C | 0.77 | 0.04 | 0.73 | 0.80 |
| rs16859382 | 2 | 219402100 | C/T | 0.81 | 0.00 | 0.81 | 0.53 |
| rs4684404 | 3 | 412533 | C/T | 0.72 | 0.08 | 0.64 | 0.84 |
| rs6442608 | 3 | 16563277 | C/T | 0.81 | 0.13 | 0.68 | 0.45 |
| rs9825091 | 3 | 56771066 | T/C | 0.85 | 0.10 | 0.75 | 0.32 |
| rs12489482 | 3 | 106062098 | A/G | 0.90 | 0.05 | 0.85 | **0.05** |
| rs7645419 | 3 | 141357284 | C/A | 0.81 | 0.02 | 0.79 | 0.97 |
| rs6802083 | 3 | 176884408 | G/T | 0.92 | 0.14 | 0.78 | 0.66 |
| rs4865470 | 4 | 1740581 | T/C | 0.80 | 0.07 | 0.73 | ***0.02*** |
| rs9291432 | 4 | 21589601 | C/T | 0.75 | 0.03 | 0.72 | 0.38 |
| rs7695466 | 4 | 52787034 | C/G | 0.84 | 0.00 | 0.84 | **0.01** |
| rs1712373 | 4 | 82374333 | A/G | 0.86 | 0.00 | 0.86 | 0.50 |
| rs159458 | 4 | 112195349 | A/G | 0.75 | 0.07 | 0.68 | 0.83 |
| rs2635256 | 4 | 149076403 | C/A | 0.84 | 0.03 | 0.81 | **0.03** |
| rs2217849 | 4 | 175238599 | A/C | 0.84 | 0.01 | 0.83 | 0.16 |
| rs9637838 | 5 | 873185 | T/C | 0.87 | 0.09 | 0.78 | 0.24 |
| rs230066 | 5 | 13576834 | A/G | 0.95 | 0.13 | 0.82 | 0.99 |
| rs4869577 | 5 | 38254937 | C/G | 0.90 | 0.08 | 0.82 | 0.39 |
| rs4296785 | 5 | 75746539 | T/C | 0.89 | 0.19 | 0.70 | 0.97 |
| rs4631225 | 5 | 108840383 | G/A | 0.87 | 0.12 | 0.75 | 0.76 |
| rs261532 | 5 | 138929546 | T/G | 0.99 | 0.24 | 0.75 | 0.17 |
| rs12522225 | 5 | 167205850 | C/A | 0.97 | 0.25 | 0.72 | 0.26 |
| rs736864 | 6 | 131221 | A/C | 0.83 | 0.21 | 0.62 | 0.11 |
| rs7770341 | 6 | 16276393 | C/A | 0.75 | 0.04 | 0.71 | 0.17 |
| rs10948308 | 6 | 46857996 | G/C | 0.82 | 0.05 | 0.77 | 0.70 |
| rs197870 | 6 | 95613585 | A/G | 0.80 | 0.06 | 0.74 | 0.36 |
| rs2286453 | 6 | 132039491 | C/A | 0.82 | 0.15 | 0.67 | 0.08 |
| rs1017643 | 6 | 156835825 | G/A | 0.99 | 0.29 | 0.70 | 0.73 |
| rs6948971 | 7 | 1820789 | G/A | 0.90 | 0.20 | 0.70 | 0.29 |
| rs4383884 | 7 | 19076229 | A/G | 0.80 | 0.03 | 0.77 | 0.18 |
| rs10264353 | 7 | 43287602 | G/A | 0.98 | 0.23 | 0.75 | ***0.002*** |
| rs1322849 | 7 | 81919475 | T/C | 0.82 | 0.17 | 0.65 | 0.79 |
| rs344454 | 7 | 146032367 | A/G | 1.00 | 0.04 | 0.96 | **0.05** |
| rs11995302 | 8 | 313502 | A/G | 0.95 | 0.16 | 0.79 | 0.48 |
| rs425010 | 8 | 17960119 | T/A | 0.77 | 0.03 | 0.74 | 0.58 |
| rs2923419 | 8 | 42585573 | G/A | 0.95 | 0.09 | 0.86 | 0.64 |
| rs1375781 | 8 | 84135108 | C/T | 0.83 | 0.13 | 0.70 | 0.99 |
| rs16932430 | 9 | 334332 | G/T | 0.94 | 0.01 | 0.93 | 0.82 |
| rs4741453 | 9 | 15075778 | A/G | 0.80 | 0.06 | 0.74 | 0.26 |
| rs10758409 | 9 | 36859256 | C/T | 0.94 | 0.27 | 0.67 | 0.99 |
| rs774227 | 9 | 93233762 | G/A | 0.89 | 0.26 | 0.63 | 0.25 |
| rs4144636 | 9 | 118802528 | T/G | 0.92 | 0.20 | 0.72 | 0.62 |
| rs816627 | 10 | 608409 | T/C | 0.85 | 0.07 | 0.78 | 0.33 |
| rs12262718 | 10 | 17383712 | A/G | 0.94 | 0.08 | 0.86 | 0.67 |
| rs10761587 | 10 | 52319199 | T/C | 0.87 | 0.14 | 0.73 | 0.31 |
| rs946714 | 10 | 80567470 | T/A | 0.78 | 0.03 | 0.75 | 0.28 |
| rs11195536 | 10 | 113101768 | G/A | 0.85 | 0.17 | 0.68 | 0.15 |
| rs7935419 | 11 | 668973 | C/T | 0.67 | 0.12 | 0.55 | 0.21 |
| rs4378393 | 11 | 21560867 | A/G | 0.86 | 0.07 | 0.79 | 0.34 |
| rs4752805 | 11 | 47974931 | G/A | 0.98 | 0.16 | 0.82 | 0.45 |
| rs10792837 | 11 | 85583363 | G/A | 0.89 | 0.13 | 0.76 | **0.03** |
| rs7934726 | 11 | 113415486 | C/T | 0.86 | 0.03 | 0.83 | 0.54 |
| rs9551445 | 13 | 18884472 | T/C | 0.75 | 0.04 | 0.71 | 1.00 |
| rs1078421 | 13 | 38577678 | C/T | 0.81 | 0.22 | 0.69 | 0.97 |
| rs9582807 | 13 | 103578174 | G/T | 0.86 | 0.07 | 0.79 | 0.45 |
| rs1620265 | 14 | 20092673 | T/G | 0.75 | 0.02 | 0.73 | 0.54 |
| rs1956424 | 14 | 36601653 | A/G | 0.73 | 0.04 | 0.69 | 0.17 |
| rs2144061 | 14 | 66596315 | C/T | 0.76 | 0.13 | 0.63 | 0.89 |
| rs6606825 | 15 | 20614243 | C/A | 0.93 | 0.14 | 0.79 | 0.09 |
| rs8035530 | 15 | 36084358 | T/C | 0.79 | 0.02 | 0.77 | 0.48 |
| rs896999 | 15 | 66887631 | G/A | 0.75 | 0.03 | 0.72 | 0.50 |
| rs977681 | 15 | 89583263 | A/T | 0.93 | 0.17 | 0.76 | 0.19 |
| rs4784375 | 16 | 52953863 | C/T | 0.92 | 0.14 | 0.78 | 0.30 |
| rs2287975 | 16 | 78107568 | G/A | 0.93 | 0.21 | 0.72 | **0.04** |
| rs7208188 | 17 | 113034 | T/C | 0.91 | 0.11 | 0.80 | 0.91 |
| rs8080590 | 17 | 14171999 | C/G | 0.79 | 0.09 | 0.70 | 0.37 |
| rs2430522 | 17 | 67204284 | G/T | 0.69 | 0.03 | 0.66 | 1.00 |
| rs568273 | 18 | 600157 | A/G | 0.68 | 0.00 | 0.68 | 0.29 |
| rs456908 | 18 | 11308729 | C/G | 0.86 | 0.21 | 0.65 | 0.97 |
| rs647853 | 18 | 38442742 | G/T | 0.98 | 0.18 | 0.80 | 0.73 |
| rs17061875 | 18 | 61973673 | A/G | 0.77 | 0.06 | 0.71 | 0.82 |
| rs2242143 | 19 | 16162544 | G/C | 0.98 | 0.28 | 0.70 | 0.80 |
| rs7255770 | 19 | 50531860 | A/G | 0.79 | 0.03 | 0.76 | 1.00 |
| rs2077713 | 20 | 166783 | C/T | 0.75 | 0.09 | 0.66 | 0.77 |
| rs768538 | 20 | 49467099 | C/A | 0.90 | 0.23 | 0.67 | 0.53 |
| rs722098 | 21 | 15607469 | G/A | 0.88 | 0.15 | 0.73 | 0.93 |
| ^a^major/minor allele assignment based on West African frequencies. Major allele frequency is given.  **^b^**delta = allele frequency difference between West African and European populations. **^c^**p-value for the association with prostate cancer risk. **Bold:** p<0.05. *Italics*: p<0.05 after correction for population stratification using STRUCTURE/STRAT. | | | | | | | |
