# Supplementary material for: Prostate Cancer Susceptibility Loci Identified on Chromosome 12 in African Americans
Source: PLoS One. 2011 Feb 16;6(2):e16044. doi: 10.1371/journal.pone.0016044 (PMC3040176; doi:10.1371/journal.pone.0016044)
Supplement: Table S4 — Fifty-five SNPs typed in previously associated or candidate chromosome 12 regions. (DOC) [file pone.0016044.s004.doc]

| Table S4. Fifty-five SNPs typed in previously associated or candidate chromosome 12 regions. | | | | | | | |
| --- | --- | --- | --- | --- | --- | --- | --- |
| **SNP id** | **chromosomal position (bp)** | **gene** | **major/minor alleles^a^** | **cases** | **controls** | **crude**  **p-value** | **gene region** |
| *rs241966* | 3710226 | EFCAB4B/FLJ33046 | C/T | 0.38 | 0.36 | 0.50 | intron |
| rs12310251 | 29604895 | TMTC1 | T/G | 0.30 | 0.33 | 0.30 | intron |
| rs299487 | 29608184 | TMTC1 | G/A | 0.28 | 0.23 | 0.09 | intron |
| rs159692 | 29623215 | TMTC1 | G/T | 0.51 | 0.46 | 0.09 | intron |
| rs11834291 | 29626903 | TMTC1 | A/T | 0.38 | 0.37 | 0.58 | intron |
| rs2113879 | 29627747 | TMTC1 | G/A | 0.52 | 0.52 | 0.94 | H318H |
| rs16934556 | 29632074 | TMTC1 | G/A | 0.22 | 0.21 | 0.71 | intron |
| rs2766605 | 29653828 | TMTC1 | G/A | 0.10 | 0.08 | 0.39 | intron |
| rs12822100^b^ | 29655403 | TMTC1 | C/G | 0.12 | 0.16 | 0.15 | intron |
| rs7976832 | 29668140 | TMTC1 | A/G | 0.24 | 0.22 | 0.65 | intron |
| rs10843457 | 29681666 | TMTC1 | T/C | 0.35 | 0.32 | 0.32 | intron |
| rs6487842 | 29709471 | TMTC1 | T/C | 0.22 | 0.24 | 0.66 | intron |
| rs7316021 | 29719889 | TMTC1 | C/T | 0.10 | 0.09 | 0.37 | intron |
| rs1863223 | 29740582 | TMTC1 | C/A | 0.30 | 0.31 | 0.62 | intron |
| rs302322 | 29819742 | TMTC1 | T/C | 0.30 | 0.28 | 0.58 | 5’UTR |
| *rs870431^c^* | 37239340 | - | A/G | 0.17 | 0.16 | 0.66 | intergenic |
| rs7968829 | 64505399 | HMGA2 | C/G | 0.00 | 0.00 | n/a | intron |
| *rs2446768* | 64525984 | HMGA2 | G/C | 0.34 | 0.33 | 0.91 | intron |
| *rs343087^b,c^* | 64547191 | HMGA2 | A/G | 0.21 | 0.18 | 0.16 | intron |
| rs1563834 | 64584298 | HMGA2 | A/G | 0.55 | 0.58 | 0.37 | intron |
| *rs1480474* | 64613210 | HMGA2 | A/G | 0.43 | 0.39 | 0.27 | intron |
| *rs10506473* | 64622005 | HMGA2 | T/A | 0.04 | 0.03 | 0.76 | intron |
| *rs17101935* | 64634647 | HMGA2 | C/A | 0.09 | 0.09 | 0.92 | intron |
| ***rs17179670*** | ***64636079*** | ***HMGA2*** | ***A/G*** | ***0.07*** | ***0.04*** | ***0.03*** | ***intron 4*** |
| *rs1351394* | 64638093 | HMGA2 | C/T | 0.40 | 0.43 | 0.40 | intron |
| *rs867633^b^* | 64641178 | HMGA2 | C/T | 0.32 | 0.33 | 0.76 | intron |
| rs2279744 | 67488847 | MDM2 | T/G | 0.13 | 0.11 | 0.22 | intron |
| rs34404568 | 78209945 | SYT1 | T/C | 0.02 | 0.03 | 0.71 | intron |
| rs7972950 | 78214103 | SYT1 | C/T | 0.00 | 0.00 | n/a | R200X |
| rs12812916 | 78366840 | SYT1 | G/T | 0.02 | 0.01 | 0.55 | V358V |
| rs11832979 | 78476928 | LOC338756 | T/G | 0.00 | 0.00 | n/a | V116G |
| *rs2307220* | 78510005 | PAWR | G/T | 0.20 | 0.21 | 0.89 | 3’UTR |
| **rs8176908** | **78510536** | **PAWR** | **G/T** | **0.09** | **0.06** | **0.03** | **V335V** |
| rs7971526 | 78518219 | PAWR | A/C | 0.007 | 0.004 | 0.55 | intron |
| **rs8176882** | **78531322** | **PAWR** | **G/C** | **0.04** | **0.02** | **0.02** | **intron 4** |
| *rs8176881* | 78537499 | PAWR | T/C | 0.03 | 0.02 | 0.10 | intron |
| *rs8176875* | 78538319 | PAWR | C/T | 0.44 | 0.39 | 0.12 | intron |
| *rs8176851* | 78542611 | PAWR | C/T | 0.03 | 0.05 | 0.12 | intron |
| *rs8176842* | 78555102 | PAWR | C/G | 0.02 | 0.02 | 0.92 | intron |
| rs8176830 | 78561079 | PAWR | C/T | 0.04 | 0.03 | 0.41 | intron |
| rs11114201 | 78563310 | PAWR | G/A | 0.02 | 0.02 | 0.64 | intron |
| rs11834585 | 78564076 | PAWR | C/T | 0.07 | 0.04 | 0.08 | intron |
| rs11114203 | 78564759 | PAWR | C/A | 0.10 | 0.07 | 0.18 | intron |
| rs2030899 | 78568058 | PAWR | C/G | 0.08 | 0.07 | 0.46 | intron |
| rs7134813 | 78579363 | PAWR | G/A | 0.33 | 0.29 | 0.14 | intron |
| *rs12316084* | 78581266 | PAWR | C/T | 0.56 | 0.54 | 0.49 | intron |
| *rs2400546* | 78588497 | PAWR | T/A | 0.34 | 0.28 | 0.09 | intron |
| rs2463169 | 78603179 | PAWR | A/G | 0.19 | 0.17 | 0.21 | intron |
| rs8176807 | 78607527 | PAWR | T/C | 0.00 | 0.00 |  | intron |
| rs8176806 | 78607746 | PAWR | G/C | 0.00 | 0.00 |  | G137A |
| ***rs12827748*** | ***78612709*** | ***PAWR*** | ***T/C*** | ***0.18*** | ***0.11*** | ***0.01*** | ***upstream*** |
| rs2694657 | 78714781 | PPP1R12A | G/C | 0.52 | 0.52 | 0.95 | L791L |
| rs12820960 | 78715196 | PPP1R12A | T/G | 0.00 | 0.00 |  | K734N |
| *rs11834113* | 92490349 | SOCS2 | C/T | 0.09 | 0.09 | 0.90 | intron |
| *rs3782415* | 92491886 | SOCS2 | A/G | 0.11 | 0.13 | 0.26 | intron |
| ^a^major/minor allele assignment based on frequencies found in West Africans. Minor allele frequency is given. ^b^HWD. ^c^SNP present in initial set of 21 AIMs. *Italics*: SNPs typed in first stage of finer mapping. **Bold**: SNPs associated with PCa, p<0.05. | | | | | | | |
